# Regulation of Sirt1/Nrf2/TNF-α signaling pathway by luteolin is critical to attenuate acute mercuric chloride exposure induced hepatotoxicity

**DOI:** 10.1038/srep37157

**Published:** 2016-11-17

**Authors:** Daqian Yang, Xiao Tan, Zhanjun Lv, Biying Liu, Ruiqi Baiyun, Jingjing Lu, Zhigang Zhang

**Affiliations:** 1 College of Veterinary Medicine, Northeast Agricultural University, 59 Mucai Street, Harbin, 150030, China

## Abstract

Inorganic mercury, though a key component of pediatric vaccines, is an environmental toxicant threatening human health *via* accumulating oxidative stress in part. Luteolin has been of great interest because of its antiinflammatory, anticarcinogenic and antioxidative effects. Here we hypothesized that luteolin would attenuate hepatotoxicity induced by acute inorganic mercury exposure. Kunming mice were treated with luteolin (100 mg/kg) 24 h after administration of 4 mg/kg mercuric chloride (HgCl_2_). The results showed that luteolin ameliorated HgCl_2_ induced anemia and hepatotoxicity, regulating radical oxygen species (ROS) production and hepatocyte viability *in vitro* and oxidative stress and apoptosis *in vivo*. Furthermore, luteolin reversed the changes in levels of inflammation- and apoptosis-related proteins involving NF-κB, TNF-α, Sirt1, mTOR, Bax, p53, and Bcl-2, and inhibited p38 MAPK activation. Luteolin enhanced antioxidant defense system based on Keap1, Nrf2, HO-1, NQO1, and KLF9. Moreover, luteolin did not affect miRNA-146a expression. Collectively, our findings, for the first time, elucidate a precise mechanism for attenuation of HgCl_2_-induced liver dysfunction by dietary luteolin *via* regulating Sirt1/Nrf2/TNF-α signaling pathway, and provide a foundation for further study of luteolin as a novel therapeutic agent against inorganic mercury poisoning.

Inorganic mercury is a well-known environmental toxicant and normally occurs in rocks, soil, water, atmosphere, and organisms in trace amounts. Researches have shown that, in mammals, mercury can induce a wide range of adverse effects on systems and tissues^1^,^2^,^3^,^4^,^5^. Aplastic anemia is another potential consequence of inorganic mercury exposure^6^. As a critical organ for drug metabolism, the liver is primary target of toxic chemicals. In chronic poisoning experiments, inorganic mercury induces severe liver injury as shown by hepatic morphological changes and apoptosis, as well as negative effect on hepatic function^5^.

The toxicity of inorganic mercury primarily involves undermining antioxidant defense systems through reactions with cellular thiols^7^. Moreover, the toxicity and therapeutic effects on some diseases of inorganic mercury have been, in part, attributed to increased oxidative stress. Mercury is, in addition, a potent apoptosis inducer, through cytochrome c release^8^, and can upregulate nuclear factor kappa-light-chain-enhancer of activated B cells (NF-κB) level^9^ and activate p38 mitogen-activated protein kinases (MAPK)^10^.

Thimerosal is regarded as an irreplaceable ingredient in some pediatric vaccines but, though it is not conclusive, is also thought to contribute to adverse neurobehavioral effects^11^. Inorganic mercury is also used in cosmetics for skin whitening^12^. In addition, daily mercury intake can occur by eating certain foods, especially fish contaminated with inorganic mercury^13^ and inhaling air which contains vapor phase and particulate mercury^14^. Overall, human health is being seriously threatened by mercury exposure.

In the clinic, using sodium 2,3-dimercapto-1-propanesulfonate as a chelating agent is an effective current treatment for removing mercuric ion (Hg^2+^) from organs^15^. Combination therapies with chelating agents, plasma exchange, hemodialysis, and plasmapheresis are used for effectively treating severe inorganic mercury poisoning^16^. Nonetheless, these therapies require long-term treatment and meticulous supportive care. Unfortunately, these treatments currently applied in the clinic are ineffective at repairing tissue damage. Considering that inorganic mercury depresses antioxidant defense system, antioxidant compounds have been proposed as potential treatments which are nontoxic and have low side effects.

Luteolin (3′,4′,5,7-tetrahydroxyflavone), a natural flavone derived from many traditional Chinese medicinal plants, has numerous health benefits. This molecule has received extensive attention because of its antiinflammatory^17^, antioxidative^18^, and anticarcinogenic^19^ activities. Increasing evidence has indicated that luteolin might modulate the homeostasis between oxidants and antioxidants, and reduce reactive oxygen species (ROS) production and apoptosis^18^. Liver, intestine, and kidney are vital target organs for luteolin. Researches have demonstrated protective effects of luteolin against liver injury induced by acetaminophen^20^ or tetrachloromethane^21^, through mechanisms involving restoring antioxidant enzyme activities and attenuating proinflammatory factors expression. Systemic administration of luteolin suppressed tumor cell growth in cancers^22^. It was proposed that luteolin ameliorated diet-induced obesity and related complications through interactions between liver and adipose tissue^23^.

The molecular mechanisms of the anticancer effects of luteolin have been well described, primarily involving cell cycle block and apoptosis inhibition^24^. However, existing literature has not supported antioxidant effect of luteolin. Nuclear factor (erythroid-derived 2)-like 2 (Nrf2) was described as being important for the antioxidant response^25^, antiinflammatory response^26^, and cytoprotection of hepatocytes^27^. A recent study showed that miRNA-146a regulated Nrf2 translation through binding to Nrf2 mRNA in aging^28^. Sirtuin type 1 (Sirt1), a NAD^+^-dependent histone deacetylase strongly expressed in the liver, is intimately related to cell proliferation, differentiation, apoptosis, and metabolism^29^. Mammalian target of rapamycin (mTOR) is also involved in drug metabolism in the liver^30^. Modulation of these factors may explain mechanisms of liver injury and hepatoprotection.

There has been increasing attention on using natural products to prevent and cure diseases induced by environmental toxicants^19^,^31^,^32^,^33^. Luteolin was reported to inhibit vascular endothelial growth factor release from human mast cells exposed to mercuric chloride (HgCl_2_)^34^. However, whether luteolin could affect hepatotoxicity induced by acute inorganic mercury exposure has not yet been elucidated. Thus, we hypothesized that luteolin would attenuate hepatotoxicity induced by acute HgCl_2_ poisoning. To address this problem, we investigated effects of dietary luteolin on HgCl_2_ induced changes in proinflammatory factors, the antioxidant defense system, and apoptotic signaling pathway as well as potential mechanisms for luteolin-mediated protection against HgCl_2_-induced hepatotoxicity.

## Results

### Protection by luteolin against HgCl_2_-induced changes in the blood

Amounts for white blood cells (WBC) and neutrophils were decreased significantly in the HgCl_2_-treated group (*P * < 0.05), whereas these were restored by luteolin (Table 1). Table 1 also showed the decreases in red blood cell (RBC) amount and hemoglobin concentration (HGB) with HgCl_2_ administration, and luteolin reversed these effects. Noticeable decreases in erythrocyte mean corpuscular volume (MCV), mean corpuscular hemoglobin (MCH), and blood platelets (PLT) amount were also observed (*P * < 0.05), while mean corpuscular hemoglobin concentration (MCHC) and red blood cell distribution width (RDW) were increased in the HgCl_2_-treated group. These findings suggested normal pigment positive cell anemia in mice. Combined with the clinical manifestations and decreased neutrophils, aplastic anemia was identified in the HgCl_2_ group. Based on the comprehensive analysis of MCV, MCH, MCHC, RDW and PLT, luteolin alleviated HgCl_2_-induced aplastic anemia.

### Luteolin attenuated HgCl_2_-induced oxidative stress in liver tissue and liver dysfunction

**Figure 1:**
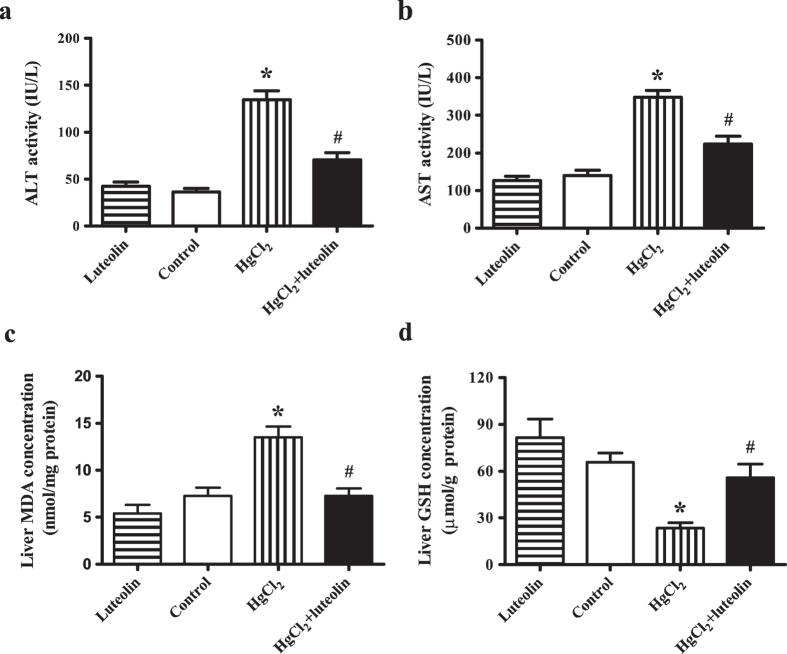
Effects of luteolin on the liver function indicators activities and oxidative stress indicators levels regulated by HgCl_2_. (**a**) ALT and (**b**) AST activities in serum of all samples from luteolin group, control group, HgCl_2_ group, and HgCl_2_ + luteolin group were detected with a Uni Cel DxC Synchron chemistry system. Values are mean ± SEM (n = 7). (**c**) MDA and (**d**) GSH concentrations in mice liver of all samples from luteolin group, control group, HgCl_2_ group, and HgCl_2_ + luteolin group were determined by commercial assay kits. Values are mean ± SEM (n = 7). *Significantly different from the corresponding control group, *P* < 0.05; ^#^Significantly different from the corresponding HgCl_2_ group, *P* < 0.05.

Alanine aminotransferase (ALT) and aspartate aminotransferase (AST) activities were used to assess liver dysfunction. ALT and AST activities in serum from different groups were shown in Fig. 1a,b. In the HgCl_2_-treated group, ALT (Fig. 1a) and AST (Fig. 1b) activities were significantly increased compared with in the control group (*P * < 0.05). However, post treatment with luteolin significantly (*P * < 0.05) reversed the effects of HgCl_2_ on serum ALT and AST activities (Fig. 1a,b).

Malondialdehyde (MDA) is a biomarker for oxidative stress and reduced glutathione (GSH) is an antioxidant, preventing damage caused by free radicals, lipid peroxide, and heavy metals. Treatment with HgCl_2_ increased MDA concentrations in liver tissue, but luteolin reversed this effect (*P * < 0.05) (Fig. 1c). GSH concentrations were clearly decreased in hepatic homogenates of the HgCl_2_-treated group, compared with the control group (*P * < 0.05), while post treatment with luteolin caused a significant increase in GSH (*P * < 0.05) compared with the levels observed in the group receiving only HgCl_2_ (Fig. 1d).

### Luteolin ameliorated HgCl_2_-induced liver injury and apoptosis

**Figure 2:**
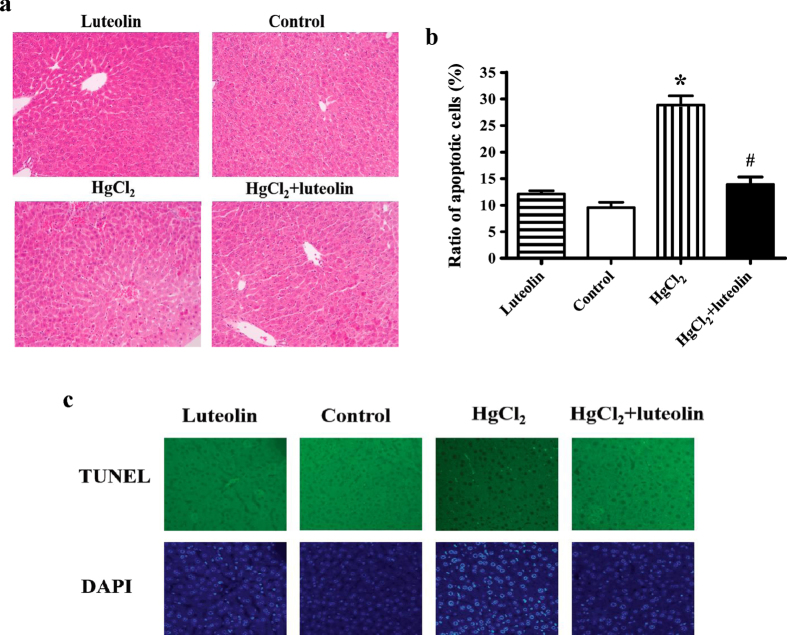
Effects of luteolin on histopathology variation and apoptosis in hepatic tissues induced by HgCl_2_. (**a**) Histopathology variation in hepatic tissue and the protective role of luteolin. Paraffin sections of hepatic tissues from luteolin group, control group, HgCl_2_ group, and HgCl_2_ + luteolin group were stained with hematoxylin-eosin (200×). (**b**) The ratio of apoptosis cells analyzed by Image J program (National Institutes of Health, Bethesda, MA, USA) was shown. Values are mean ± SEM (n = 7). *(**c**) Representative images of tissues from mice treated with luteolin, no medicine, HgCl_2_, and HgCl_2_ combined with luteolin. TUNEL-positive cells were showed. *Significantly different from the corresponding control group, *P* < 0.05; ^#^Significantly different from the corresponding HgCl_2_ group, *P* < 0.05.

Histopathological assessments of liver sections from the mice were shown in Fig. 2a. Congestion of the central vein was observed, with severe erythrocyte infiltration, in the HgCl_2_-treated group, along with plasmolysis of the hepatocytes and broadening of the hepatocellular gap around the central vein. In the HgCl_2_ + luteolin group, there was swelling of hepatocytes and slight erythrocyte infiltration. However, there were no obvious histopathological changes in livers from the other groups.

As shown by the TUNEL assay (Fig. 2c), in the HgCl_2_-treated group, the level of apoptotic hepatocytes (Fig. 2b) was significantly higher than in the control group. However, there was no significant difference between luteolin and control group. The stimulatory effect by HgCl_2_ was attenuated in the HgCl_2_ + luteolin-treated group, indicating that luteolin significantly prevented HgCl_2_-induced apoptosis (*P * < 0.05).

### Luteolin reversed the changes in protein levels regulated by HgCl_2_

**Figure 3:**
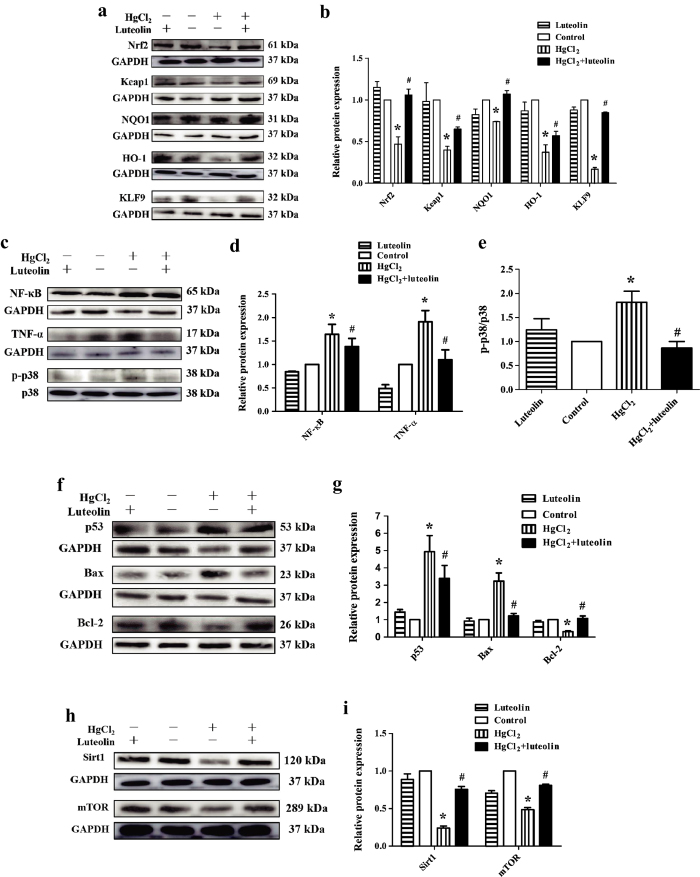
Effects of luteolin on protein levels changed by HgCl_2_. (**a**) Western blot analysis of Nrf2 and Nrf2-related protein levels in the liver. Anti-GAPDH antibody was used as a loading control. (**b**) Quantified protein levels were shown. Uncropped images are provided in Supplementary Figure 2A. (**c**) Western blot analysis of NF-κB, TNF-α, p-p38 and p38 in the liver. Anti-GAPDH antibody was used as a loading control in measurements of NF-κB and TNF-α. Anti-p38 antibody was used as a loading control in measurement of p38 activation. Uncropped images are provided in Supplementary Figure 2B. (**d**) Quantified protein levels of NF-κB, TNF-α were shown. (**e**) The ratio of p-p38 was shown. (**f**) Western blot analysis of p53, Bax and Bcl-2. Anti-GAPDH antibody was used as a loading control. Uncropped images are provided in Supplementary Figure 2C. (**g**) Quantified protein levels were shown. (**h**) Western blot analysis of Sirt1 and mTOR. Anti-GAPDH antibody was used as a loading control. Uncropped images are provided Supplementary Figure 2D. (**I**) Quantified protein levels were shown. Values are mean ± SEM (n = 4). Each two blots were run under the same conditions.*Significantly different from the corresponding control group, *P* < 0.05; ^#^Significantly different from the corresponding HgCl_2_ group, *P* < 0.05.

The complex of Nrf2 and Kelch-like ECH-associated protein 1 (Keap1) plays major role in modulating cellular oxidative stress. We found that Nrf2 and Keap1 were involved in protection against HgCl_2_-stimulated oxidative stress by luteolin. HgCl_2_ induced a significant (*P * < 0.05) decrease in translation of Nrf2 and Keap1, and luteolin significantly (*P * < 0.05) reversed these effects. Levels of heme oxygenase 1 (HO-1) and NAD(P)H: quinone oxidoreductase 1 (NQO1), target proteins of Nrf2, were significantly (*P * < 0.05) lower in the HgCl_2_-treated than in the control group. However, luteolin post treatment significantly attenuated this decrease (*P * < 0.05). In addition, it was reported that oxidative stress increased Kruppel-like factor 9 (KLF9) level^35^. In our study, level of KLF9 was significantly (*P * < 0.05) decreased in the HgCl_2_-treated group, compared with control group, and luteolin significantly (*P * < 0.05) reversed this effect (Fig. 3a,b).

Levels of NF-κB and tumor necrosis factor alpha (TNF-α) were significantly higher in the HgCl_2_-treated than in the control group (*P * < 0.05). In Fig. 3c–e, luteolin alone decreased NF-κB and TNF-α levels in the liver, and it also significantly (*P * < 0.05) prevented NF-κB expression and TNF-α production induced by HgCl_2_. Treatment with HgCl_2_ significantly enhanced phosphorylation of p38 MAPK (*P * < 0.05), but luteolin produced a significant (*P * < 0.05) inactivation of p38 MAPK against active action of HgCl_2_.

The levels of B-cell lymphoma 2 (Bcl-2), a prosurvival protein, was significantly decreased with HgCl_2_ treatment (*P * < 0.05). However, levels of the proapoptotic proteins including Bcl-2-associated X protein (Bax) and p53 were significantly increased in the HgCl_2_-treated group (*P * < 0.05). In contrast, with luteolin treatment, there were significant upregulation of Bcl-2 and suppression of Bax and p53 (*P * < 0.05) (Fig. 3f,g).

Sirt1 and mTOR are proteins related to metabolism. Sirt1 and mTOR levels were significantly (*P * < 0.05) suppressed in the HgCl_2_-treated group, but luteolin reversed this effect (Fig. 3h,i).

### HgCl_2_ and luteolin had no effect on miRNA-146a expression

**Figure 4:**
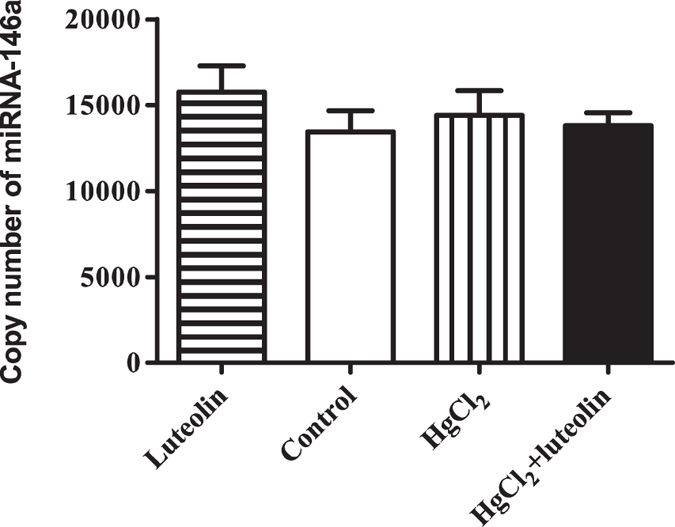
Effects of luteolin and HgCl_2_ on expression miRNA-146a. Luteolin and mercury had no effect on miRNA-146a transcription which targets Nrf2 in liver. Liver from different groups were collected and absolute quantitative realtime RT-PCR analysis for miR-146a was performed. The copy number of miRNA-146a of different groups was shown. All results were representative of 4 independent experiments, each performed in triplicate. Values are mean ± SEM. No significant differences were observed.

MiRNA-146a is a potential regulator of Nrf2^28^. As shown in Fig. 4, no significant differences in miRNA-146a levels were observed among the 4 groups. Neither HgCl_2_ nor luteolin affected miRNA-146a transcription in this experiment.

### Luteolin enhanced hepatocyte viability and attenuated ROS levels induced by HgCl_2_

**Figure 5:**
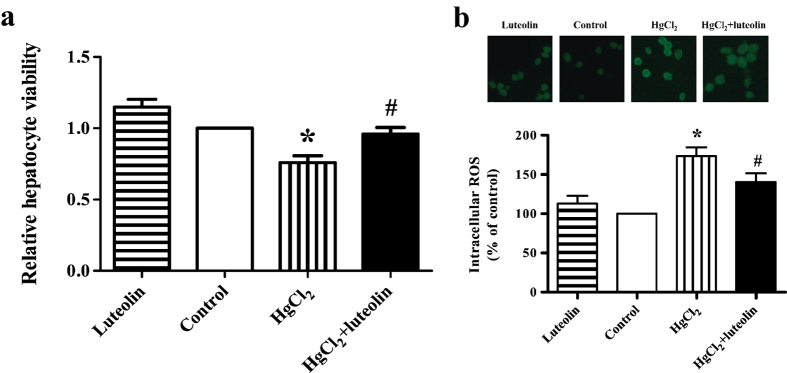
Inhibition of luteolin on HgCl_2_-induced cytotoxicity and ROS production in hepatocytes. (**a**) Cell viability of primary hepatocytes in mice with different treatments was detected by CCK-8 kit. Primary hepatocytes were grown in 96-well plates at a density of 10^4^ cells per well and cultured overnight. Hepatocytes were treated with 5 μM HgCl_2_ for 24 h, with or without pretreatment with 20 μM luteolin for 2 h. The medium was discarded and hepatocytes were incubated in 100 μL medium with 10 μL CCK-8 solution at 37 °C for 4 h. The optical density was measured at 450 nm on a Bio-Tek Epoch microplate reader. Values are mean ± SEM (n = 4). (**b**) Generation of intracellular ROS was determined by the Reactive Oxygen Species Assay Kit. Primary hepatocytes were grown in 6-well plates at a density of 10^4^ cells per well and cultured overnight. Hepatocytes were treated with 5 μM HgCl_2_ for 24 h, with or without pretreatment with 20 μM luteolin for 2 h. After treatments, cells were incubated with DFCH-DA at a final concentration of 10 μM at 37 °C for 20 min. Values are mean ± SEM (n = 4). *Significantly different from the corresponding control group, *P* < 0.05; ^#^Significantly different from the corresponding HgCl_2_ group, *P* < 0.05.

As shown in Fig. 5a, 5 μM HgCl_2_ significantly decreased cell viability (*P * < 0.05) in comparison with the control group. However, treatment of cells with 20 μM luteolin alone increased hepatocyte viability and pretreatment with 20 μM luteolin significantly reversed the effects of HgCl_2_ on cell viability (*P * < 0.05). Moreover, Fig. 5b illustrated that HgCl_2_ significantly enhanced ROS level in hepatocytes (*P * < 0.05), however, this was attenuated by luteolin (*P * < 0.05).

## Discussion

Inorganic mercury is an important environmental pollutant causing systemic toxicity and threatens human health. Results of WBC count, a biomarker of chemical intake, in our study, luteolin maintains WBC amount. This suggests that luteolin attenuates HgCl_2_-induced injury possibly through attenuating total mercury accumulation in mice. Complete blood analysis indicates that there is aplastic anemia in mice treated with HgCl_2_, which is consistent with other reports^6^,^36^. Therefore, we hypothesize that HgCl_2_ might induce hemopoietic stem cell injury, while luteolin may serve a protective role in its progression. Strong evidence has suggested that inorganic mercury potently inhibits uroporphyrinogen decarboxylase^5^, an important enzyme catalyzing conversion of uroporphyrinogen to coproporphyrinogen. This can prevent heme synthesis and ultimately arrest production of hemoglobin. Luteolin most likely protects against inorganic mercury and restores hemoglobin levels by attenuating the obstruction of heme biosynthesis induced by HgCl_2_. All the above results suggest that luteolin may be useful for reducing the toxic effects of Hg^2+^ on uroporphyrinogen decarboxylase, ameliorating anemia, inhibiting Hg^2+^ accumulation, and attenuating injury to the organism.

The serum ALT and AST activities indicate that luteolin protects the mice from HgCl_2_-induced liver injury. Its protection against liver injury is consistent with the liver histological observations. Moreover, luteolin mitigates HgCl_2_-induced apoptosis and maintains hepatocyte viability. Together, all these results indicate that luteolin inhibits HgCl_2_-induced hepatic inflammation, apoptosis, and cytotoxicity.

The mechanism of liver injury induced by HgCl_2_ is believed to involve ROS production and free radical mediated damage. Measurements of MDA and GSH of HgCl_2_-treated mice indicate ROS production and free radical damage, in good agreement with the cellular ROS levels *in vitro*. Hg^2+^ complexed tightly with hydrosulphonyl moieties after entering the body, causing depletion of intracellular hydrosulphonyl moieties and release of reactive oxygen free radicals. It resulted, either indirectly or directly, in oxidative stress and lipid peroxidation^37^. However, luteolin attenuates oxidative stress and free radical damage, and enhances the antioxidant system including superoxide dismutase (SOD) and GSH, indicating that luteolin provides protection against HgCl_2_-induced lipid peroxidation and oxidative stress.

Findings above confirm that luteolin arrests ROS production and decreases oxidative stress to prevent HgCl_2_-induced hepatotoxicity. Excessive oxidative stress consumes a large amount of Nrf2 and Keap1, disrupting the homeostasis between expression and degradation of these two factors. Our protein expression data indicate that luteolin promotes Nrf2 expression, and reverses the depletion of Nrf2 caused by acute HgCl_2_ exposure, thus improves the ability to resist oxidative stress.

Levels of downstream proteins of Nrf2 such as NQO1, HO-1 and SOD were upregulated in HgCl_2_ + luteolin group in good agreement with effect of luteolin on Nrf2 levels. This indicates that luteolin activates Nrf2 signaling pathway to benefit detoxification and antioxidant defense system. KLF9 modulated cell death and oxidative injury under conditions of excessive oxidative stress, and was positively regulated by Nrf2^35^. Therefore, it can be concluded that luteolin attenuates HgCl_2_-induced oxidative stress *via* alleviating depletion of Nrf2 and activating Nrf2 signaling pathway to upregulate KLF9 and enhance antioxidant defense system.

TNF-α is a cytokine involved in systemic inflammation and a component of the acute phase reaction^38^. In this study, luteolin suppresses TNF-α production in the liver in the presence of HgCl_2_. Luteolin also was reported to inhibit TNF-α release by inhibiting extracellular regulated protein kinases, p38 MAPK, and casein kinase 2 activation from macrophages^39^. TNF-α, when binding to tumor necrosis factor receptors (TNFR), binds to the TNFR type 1-associated death domain protein (TRADD) and then activates p38 MAPK and NF-κB^40^. P38 MAPK represents a class of MAPKs that can also activate NF-κB^41^. Our results suggest that luteolin reduces NF-κB and phosphorylation of p38 which occurs in the presence of HgCl_2_. This implies that luteolin inhibits TNF-α to inactivate p38 MAPK and inhibit NF-κB, to reverse the HgCl_2_-induced inflammatory response. Nrf2 has a negative effect on TNF-α expression^42^, which suggests that upregulation of Nrf2 by luteolin may target inactivation of HgCl_2_-induced inflammatory signaling pathways. Luteolin attenuates HgCl_2_-induced excessive oxidative stress to ameliorate inflammation thereby preventing liver injury. Therefore, we conclude that Nrf2 is a key regulatory factor in antioxidant and antiinflammatory defense systems, and plays a critical role in the protection against HgCl_2_ exposure by luteolin.

Apoptosis signaling pathways involves p53 and the Bcl-2 protein family^43^, including proapoptotic and prosurvival proteins^44^. The tumor suppressor protein p53 influences apoptosis and can modulate levels of the Bcl-2 protein family^43^. In our study, luteolin suppresses p53, thereby increases Bcl-2 level and decreases Bax level, and finally protects hepatocytes against HgCl_2_-induced apoptosis.

NF-κB activation plays a dual role in regulating apoptosis in various tissues and cells^45^,^46^. The relative protein levels of Bcl-2, Bax, and NF-κB show that luteolin suppresses NF-κB, thereby inhibits apoptosis^45^. NF-κB and p53 could be upregulated by p38 MAPK^41^,^47^. Activation of the p38 pathway significantly stimulated p53 function^47^. Moreover, p38 MAPK also affected NF-κB levels by promoting phosphorylation of IκB, resulting in the dissociation and degradation of NF-κB and IκB complexes^41^. The levels of p53, NF-κB, and p38 demonstrates that luteolin inhibited p38-activated NF-κB and p53 pathways, which then contributes to the protection of luteolin against HgCl_2_-induced inflammation and apoptosis.

Sirt1, a NAD^+^-dependent protein deacetylase, regulates such cellular processes as stress response and longevity^48^. mTOR is a serine/threonine protein kinase that regulates cell survival, protein synthesis, and translation^49^. Our data, for the first time, show that luteolin activates Sirt1 and mTOR, which are inhibited by HgCl_2_. Sirt1 directly suppresses NF-κB and p53 activation, because its *N*-terminal domain promotes deacetylation of NF-κB p65^48^ and p53^50^. This suggests that, in our experiments, luteolin protects hepatocytes and inhibits the inflammation and apoptosis *via* promoting Sirt1 expression to suppress NF-κB and p53 induced by HgCl_2_ exposure. Moreover, there is reliable evidence that mTOR can regulate Bcl-2 activation, by a positive feedback mechanism, to inhibit apoptosis^51^. In addition, it was reported that Sirt1 activated the Nrf2 pathway to decrease ROS production induced by advanced glycation end products in glomerular mesangial cells^52^. Together, all these demonstrate that Sirt1 is a key factor in regulating inflammation, apoptosis and antioxidant defense systems, contributing to prevent the hepatotoxity of HgCl_2_ by luteolin.

Interestingly, miRNA-146a was reported to inhibit Nrf2 protein synthesis, but maintaining Nrf2 mRNA levels, in aging rats^28^. Luteolin was also reported to inhibit procarcinogenic miRNAs^53^. Regretfully, in our study, neither HgCl_2_ nor luteolin has any effect on miRNA-146a transcription, arguing against a role for miRNA-146a in the effects we observed with both HgCl_2_ and luteolin. Therefore, we infer that luteolin may maintain Nrf2 production in mice liver by activating the Sirt1 signaling pathway or by directly affecting the Nrf2-Keap1 complex.

**Figure 6:**
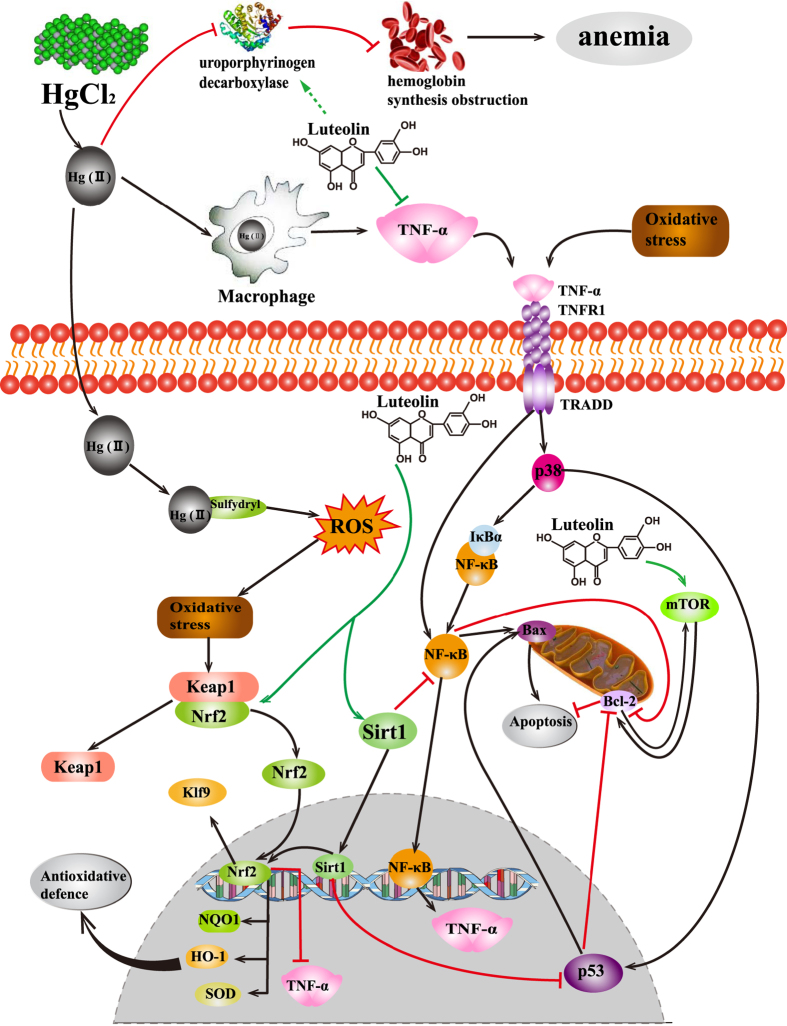
The mechanism of luteolin attenuating the toxicity in the liver and blood induced by acute HgCl_2_ exposure. *Green line* denotes stimulatory or inhibitory effect of luteolin, *black line* denotes stimulatory effect, and *red line* denotes inhibitory effect.

In conclusion, luteolin protects hepatocytes from oxidative stress, inflammation, and apoptosis induced by HgCl_2_ in the liver *via* modulating the Sirt1/Nrf2/TNF-α signaling pathway (summarized in Fig. 6). Moreover, luteolin also have attenuated HgCl_2_-induced blood toxicity by modulating hemoglobin synthesis and reducing mercury accumulation, though the detailed mechanism still requires further study. Therefore, we insist luteolin, in combination with inorganic mercury, may improve the safety of pediatric vaccines with mercury. In addition, dietary intake of luteolin may offer a novel and safe method to protect human health against inorganic mercury exposure.

## Materials and Methods

### Animals and treatments

All animal protocols were approved by the Ethical Committee for Animal Experiments (Northeast Agricultural University, Harbin, China). Twenty-eight adult healthy male Kunming mice (25 ± 5 g body weight) were obtained from Harbin Veterinary Research Institute (Harbin, China). All animals were acclimated for 1 w under the same laboratory conditions with a 12 h interval light/dark cycle, a minimum of 40% relative humidity, a room temperature of 21 ± 4 °C, standard food, and water ad libitum. Housing and experimental facilities at the Northeast Agricultural University were approved by the Chinese Ministry of Agriculture and animal care and experimental protocols conformed with the Guide for the Care and Use of Laboratory Animals (Institute of Laboratory Animal Resources, Commission on Life Sciences, National Research Council, 2000).

The 28 mice were randomly and equally divided into 4 groups of 7 animals each. The groups were: control, luteolin, HgCl_2_, and HgCl_2_ + luteolin. Total HgCl_2_ (4 mg/kg) (Beijing Chemical Plant, Beijing, China) was administered by intraperitoneal injection, as a suspension in 0.9% (w/v) physiological saline. Luteolin (100 mg/kg) (Xi’an Weiao Biological Technology Company Ltd., Xi’an, China) was administered intragastrically as a suspension in 1% (v/v) dimethyl sulfoxide (DMSO). In the control group, equal amount of 0.9% physiological saline and 1% DMSO were given as vehicles orderly. In the luteolin group, mice received a single dose of luteolin (100 mg/kg) only. In the HgCl_2_-treated group, mice received HgCl_2_ (4 mg/kg) only, and in the HgCl_2_ + luteolin-treatment group mice received luteolin (100 mg/kg) 24 h after HgCl_2_ administration.

All mice were killed by given ether anesthesia 24 h after the last treatment. Blood samples were collected from the abdominal vein into vacuum tubes containing heparin sodium anticoagulant. Liver tissues were rapidly excised and homogenized in phosphate-buffered saline (PBS) pH 7.4 using an Ultra-Turrax T25 Homogenizer. After centrifugation at 10,000 × *g* for 10 min at 4 °C, the supernatant was used for biochemical determinations.

### Complete blood count and biochemical analysis

Some of the blood samples were used for complete blood count, which were obtained with an automated Auto Hematology Analyzer BC-2600Vet (Mindray, Shenzhen, China). Other blood samples were centrifuged at 3,000 × *g* for 10 min. Activities of ALT and AST were detected in the serum with a Uni Cel DxC Synchron chemistry system (Beckman Coulter Inc., Fulton, CA, USA).

### Measurement of oxidative stress indicators in liver tissues

MDA and GSH levels in tissues were determined by commercial assay kits from Jiancheng Bioengineering Institute (Nanjing, China) according to the manufacturer’s instructions.

### Histopathology

Liver tissues from mice were fixed in 10% formaldehyde overnight at 4 °C. Tissues were cut into blocks of 3 mm thickness. Tissue blocks were then embedded in paraffin. Sections (5 μm thickness) were cut on the coronal plane and stained with hematoxylin and eosin. Morphology was examined under a light microscope (BX-FM: Olympus Corp, Tokyo, Japan).

### Terminal Deoxynucleotidyl Transferase-mediated dUTP Nick-End Labeling (TUNEL) staining assay

The TUNEL Assay Kit (Beyotime Institute of Biotechnology, Jiangsu, China) was used to assess hepatocyte apoptosis. Sections of liver tissue were placed in 50 μM TUNEL detection solution, then washed twice with PBS and incubated for 60 min at 37 °C in the dark. The sections were observed under a fluorescence microscope at an excitation wavelength range of 450–500 nm and emission wavelength range of 515–565 nm.

### Western blot analysis

The Bicinchoninic Acid Kit (Beyotime Institute of Biotechnology) was used to determine protein content of liver samples to ensure gel loading for western blots. Equal aliquot (8 μg) of the protein samples were separated by SDS-PAGE gel electrophoresis using a BioRadÓ Mini-PROTEANÒ 3 electrophoresis cell (BioRad, Hercules, CA, USA) and electrophoretically transferred to polyvinylidene fluoride membrane (Immobilon^®^-P Transfer Membrane, EMD Millipore, Billerica, MA, USA), and then the membranes were probed with appropriate combination of primary and horseradish peroxidase-conjugated secondary antibodies from Santa Cruz Biotechnology (Dallas, TX, USA). Proteins in the membranes were visualized by enhanced chemiluminescence kits. The protein bands were quantified by the average ratios of integral optic density following normalization to the levels of internal control GAPDH, and the results were further normalized to control.

### MiRNA-146a isolation and absolute quantitative real-time PCR analysis

MiRNA-146a was extracted using SanPrep Column miRNA Mini-Preps Kit (Sangon Biotech, Shanghai, China) according to the manufacturer’s instructions. MiRNA-146a detection by real-time analysis involved reverse transcription of cDNA using a small RNA specific stem-loop RT primer (mmu-miR-146a-5p; 5′-CTCAACTGGTGTCGTGGAGTCGGCAATTCAGTTGAGAACCCATGG-3′). Once specific cDNA had been generated, individual miRNA was detected using SYBR Green RNA assay real time PCR analysis (mmu-miR-146a-5p; 5′-ACACTCCAGCTGGGTGAGAACTGAATTC-3′). Real-time PCR was conducted using Roche LightCycler480 (Roche, Basel, Switzerland). The thermal cycling included 3 min of denaturation at 95 °C followed by 45 PCR cycles, including 15 s at 95 °C, 20 s at 57 °C, and 30 s at 72 °C. Linearized plasmid was quantified using a spectrophotometer and copy numbers were calculated.

### Establishment of the absolute quantitative standard curve

In order to examine the miRNA-146a copy number, generation of the absolute quantitative standard curve was necessary. Six different concentrations of standard samples were prepared respectively, by mixing cDNA obtained by reverse transcription with plasmid XM709-2. The parameters of the standard curve was: log *N* = −3.176Δ*Ct* + 36.91 (*R*^2^ = 0.9981, *P* < 0.01). The standard curve was shown in Supplementary Figure 1.

### Hepatocyte culture and treatment

Adult male Kunming mouse was injected intraperitoneally with pentobarbital and heparin. Mouse hepatocytes were prepared as described previously^54^. Briefly, the liver was perfused *in situ* with collagenase (Sigma, St. Louis, MO, USA) through the hepatic portal vein. The total cells released were centrifuged 3 times, for 3 min at 100 × *g*, 50 × *g*, 50 × *g*. Hepatocytes were suspended at a density of 5 × 10^5^ cells/mL in adherent culture medium. Dulbecco modified eagle medium (DMEM, Invitrogen, Grand Island, NY, USA) was supplemented with 2 g/L HEPES (Gibco, NY, USA), 6 mg/L insulin (Sigma), 1 mg/L dexamethasone (Sigma), 1% (v/v) penicillin/streptomycin (Thermo Fisher Scientific), and 10% (v/v) fetal bovine serum (Hyclone, Logan, UT, USA). Next, 2.5 mL cell suspension was plated into 6-well plates containing collagen-coated glass cover slips. After culturing at 37 °C under 5% CO_2_ for 24 h, medium and nonadherent hepatocytes were aspirated and replaced with culture medium containing 5% (v/v) fetal bovine serum.

### Determination of hepatocyte viability

Hepatocyte viability was determined by using WST tetrazolium salt (CCK-8, Dojindo, Kumamoto, Japan) following the manufacturer’s instructions. Briefly, 10^4^ cells/well were seeded in 96-well plates with DMEM media. After culture overnight, hepatocytes were treated with 5 μM HgCl_2_ for 24 h, with or without pretreatment with 20 μM luteolin for 2 h. The medium was discarded and hepatocytes were incubated in 100 μL medium with 10 μL CCK-8 solution at 37 °C for 4 h. The optical density was measured at 450 nm on a Bio-Tek Epoch microplate reader (Bio-Tek, Winooski, VT, USA).

### Measurement of ROS generation

Generation of intracellular ROS was determined by the Reactive Oxygen Species Assay Kit (Beyotime Institute of Biotechnology) according to the manufacturer’s instructions. Briefly, 10^4^ cells/well were seeded onto square glass coverslips (24 × 24 mm) in 6-well plates. After overnight culture, the hepatocytes were treated with 5 μM HgCl_2_ for 24 h with or without pretreatment with 20 μM luteolin for 2 h. After treatments, cells were incubated with DFCH-DA at a final concentration of 10 μM at 37 °C for 20 min. The hepatocytes were observed by fluorescence microscopy (Olympus IX51, Nikon, Tokyo, Japan) with an excitation wavelength of 488 nm and emission wavelength of 525 nm.

### Statistical analysis

Data are presented as mean ± standard error of the mean (SEM). Statistical analyses were performed with SPSS 19.0 software (SPSS, Chicago, IL, USA). Shapiro-Wilk was performed to assess the normality of the data, and Levene’s Test for equality of variances was performed. One-way analysis of variance was used to determine differences among 4 groups. Tukey Test for post hoc multiple comparison was used to determine differences between means. A two-tailed *P* < 0.05 was considered as being significant.

## Additional Information

**How to cite this article**: Yang, D. *et al*. Regulation of Sirt1/Nrf2/TNF-α signaling pathway by luteolin is critical to attenuate acute mercuric chloride exposure induced hepatotoxicity. *Sci. Rep.* **6**, 37157; doi: 10.1038/srep37157 (2016).

**Publisher’s note:** Springer Nature remains neutral with regard to jurisdictional claims in published maps and institutional affiliations.

## Supplementary Material

Supplementary Information

## Figures and Tables

**Table 1:** Complete blood cell parameters of luteolin, control, HgCl_2_, and HgCl_2_ + luteolin cases.

Index	Luteolin	Control	HgCl_2_	HgCl_2_ + luteolin
White blood cell (WBC) (×10^9^/L)	7.57 ± 0.34	7.79 ± 0.25	5.57 ± 0.23*	7.61 ± 0.29#
Neutrophils (×10^9^/L)	6.9 ± 0.29	8.01 ± 0.33	5.27 ± 0.32*	7.03 ± 0.36#
Red blood cell (RBC) (×10^9^/L)	9.28 ± 0.46	10.08 ± 0.57	8.24 ± 0.29*	9.942 ± 0.37#
Haemoglobin (HGB) (g/L)	114.29 ± 7.521	127 ± 18.34	97.14 ± 6.31*	120.86 ± 6.27#
Mean cell volume (MCV) (fL)	38.83 ± 1.03	42.19 ± 0.80	37.81 ± 0.92*	41.76 ± 0.66#
Mean cell haemoglobin (MCH) (pg)	12.3 ± 0.46	13.27 ± 0.56	11.48 ± 0.54*	12.84 ± 0.32#
Mean cell haemoglobin concentration (MCHC) (g/L)	296.57 ± 7.07	292.14 ± 9.50	325.71 ± 11.30*	293.29 ± 9.31#
Red cell distribution width (RDW) (%)	17.87 ± 0.69	15.68 ± 0.67	18.57 ± 0.63*	15.3 ± 0.64#
Platelet (PLT) (×10^9^/L)	554.43 ± 72.15	485 ± 58.90	254.86 ± 32.54*	379.83 ± 19.91#

Complete blood count was determined by an automated Auto Hematology Analyzer BC-2600Vet. Values are mean ± SEM (n = 7). ^*^Significantly different from the corresponding control group, *P* < 0.05; ^#^Significantly different from the corresponding HgCl_2_ group, *P* < 0.05.
