# Supplementary material for: Regulation of Sirt1/Nrf2/TNF-α signaling pathway by luteolin is critical to attenuate acute mercuric chloride exposure induced hepatotoxicity
Source: Sci Rep. 2016 Nov 17;6:37157. doi: 10.1038/srep37157 (PMC5112569; doi:10.1038/srep37157)
Supplement: Supplementary Information [file srep37157-s1.pdf]

Supplementary Information of

Regulation of Sirt1/Nrf2/TNF-α signaling pathway by luteolin is critical to attenuate acute mercuric chloride exposure induced hepatotoxicity

Daqian Yang, Xiao Tan, Zhanjun Lv, Biying Liu, Ruiqi Baiyun, Jingjing Lu & Zhigang Zhang*

College of Veterinary Medicine, Northeast Agricultural University, 59 Mucai Street, Harbin 150030, China

E-mail address: zhangzhigang@neau.edu.cn

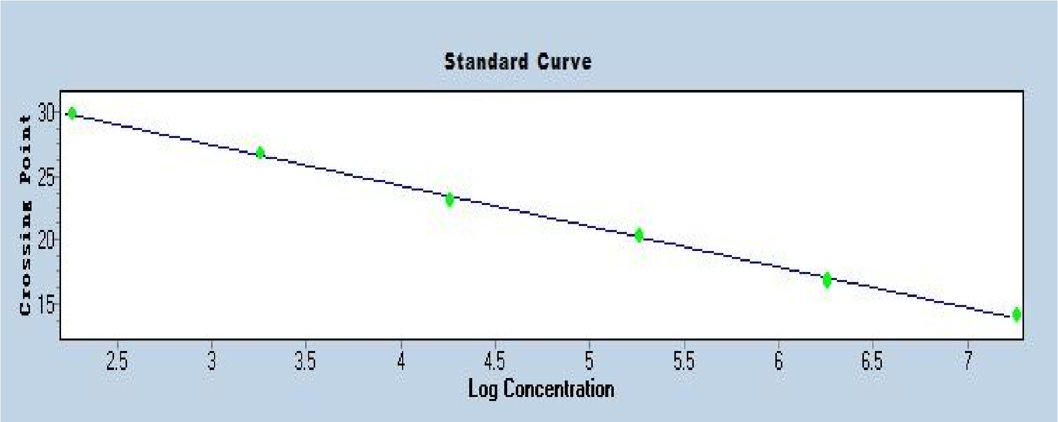

Figure S1. Standard curve of miRNA-146a was conducted using Roche LightCycler480 (Roche). The parameters of the standard curve was: $\log_2 N = -3.176\Delta Ct + 36.91 (R^2 = 0.9981, p < 0.01)$. PCR efficiency was 106.5 % and the correlation coefficient was 0.9981.

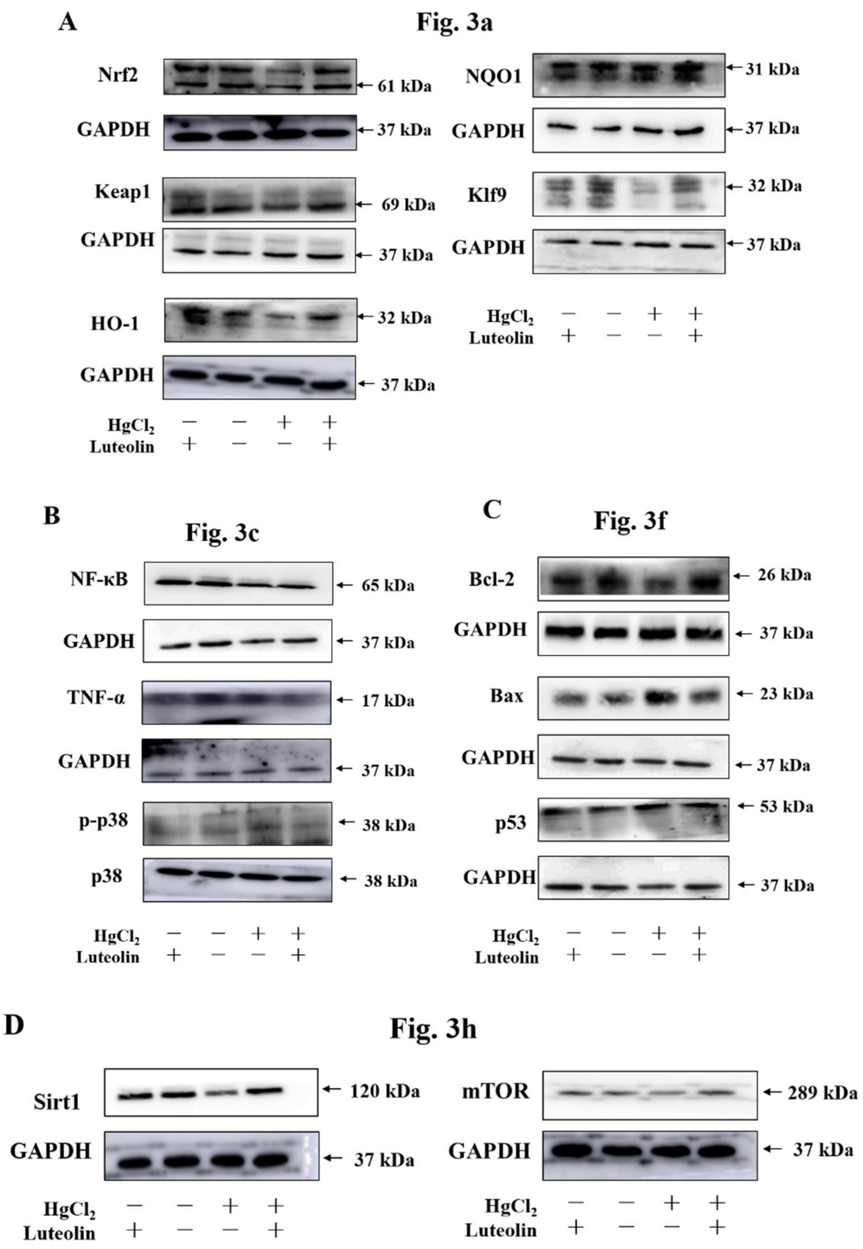

Figure S2. Original images of cropped blots.
